# Electricity in Modern Medicine

**Published:** 1913-06-21

**Authors:** Alfred C. Norman

**Affiliations:** House Surgeon at Durham County Eye Infirmary.


### ELECTRICITY IN MODERN MEDICINE.*

### XXXI.—Electricity in Ophthalmic Practice.

By ALFRED C. NORMAN, M.D. Edin., House Surgeon at Durham County Eye Infirmary.

Since the sections on "$x$-rays" appeared in The Hospital the writer has received several letters concerning $x$-ray installations suitable for small hospitals of from fifty to a hundred beds, and asking where the apparatus could be obtained at the price mentioned in the first article. The writer has had practical experience in three different institutions of outfits supplied by Messrs. Schall and Son,† and has in each instance been well satisfied with them. The following detailed list of apparatus may be of use to others who contemplate putting in new $x$-ray installations :—

Twelve-inch induction coil. New pattern Mackenzie-Davidson interrupter. Shunt-series switchboard. Two Gundelach heavy anode tubes, with automatic regulation. Tube stand with compressor diaphragm. Milliamperemeter, oscilloscope tube, and valve tube mounted on special overhead frame, with all necessary terminals for spintermeter, etc. Plain couch, with wooden top. Fluorescent screen. Wehnelt's small radiometer. Set of lead figures for numbering radiographs. Photographic accessories as follows :—$x$-ray plates, one dozen of each size, 12 by 10, 10 by 8, 8½ by 6½, and 6½ by 4¾. Dishes, printing frames, printing paper, and light tight envelopes for each size of plate. Dark-room lamp.

Such an outfit is listed at about £80, and most instrument makers give a special discount of 10 per cent. to hospitals, bringing the net cost down to £72.

### Electricity in Ophthalmic Practice.

No other branch of medicine has gained more than has ophthalmology by the rapid advances made in electrical science during the last few years. Apart from general electro-therapeutic methods which are sometimes of value in treating eye diseases, electricity is used in ophthalmic practice: (1) As a source of illumination; (2) to furnish $x$-rays; (3) to excite powerful electro-magnets; (4) for ionic medication of corneal ulcers, rodent ulcers, and lupus of the eyelids, etc.

### Electric Light in Ophthalmology.

The introduction of the incandescent lamp led to great improvements in the design and portability of many ophthalmic instruments and to the introduction of others that would have been impossible with the older forms of illumination. The ophthalmometer or keratometer, used for estimating astigmatism, has been transformed from an ungainly fixture to the neat, portable and accurate piece of apparatus now found in nearly all oculists' consulting rooms. Electric hand lamps, projectors, transilluminators, amblyoscopes, retinoscopes, ophthalmoscopes, etc., are coming more and more into general use as efficient patterns are evolved by responsible firms, but in this class of instrument much rubbish has been sold in the past. As a dark-room fixture for general purposes, retinoscopy, and indirect ophthalmoscopy, nothing is better than a 16 c.p. frosted bulb (preferably circular in shape like the Stearn lamp) mounted on a vertically sliding wall-bracket.

### The Morton-Marple Ophthalmoscope.

For direct examination of the fundus oculi the Morton-Marple electric ophthalmoscope has no equal. With this instrument it is as easy to make a detailed examination of the fundus oculi as it is to look into a room through the window. Owing to the fact that there is no trouble with the source of light, the mirror can be brought right up to the patient's eye, thus allowing the observer to look through the pupil exactly as if he were looking through a window into a darkened room, and this, of course, without employing a mydriatic. The beam of light is condensed by a small lens in the tube of the instrument, consequently it is very powerful and brings out minute details in the fundus that even an expert could not see by any other method. The instrument is also of infinite value to the novice. Men who have never before been able to use an ophthalmoscope can make out every detail of the fundus the first time they look through the Morton-Marple instrument. In fact, they acquire as much proficiency in one day as would previously have taken them three or four years with the older reflecting instrument.

The instrument consists of an ordinary Morton ophthalmoscope, the mirrors of which are replaced by a lighting attachment designed by Dr. Marple, of New York. This attachment consists of a metal tube in which are arranged a tiny electric lamp, a lens, and a U-shaped mirror. The slot of the mirror should be cut through the glass itself—a point not always attended to by the makers—in order to prevent reflection from the upper part. The lens is a fixture and the lamp below it can be moved up and down the tube by means of a button on a sliding sleeve, thus allowing the degree of convergence of the beam of light to be varied at will. To obtain the best results the user must accustom himself to moving the sliding sleeve while actually making an examination, and he will be surprised at the amount of detail that is brought out as the beam of light is gradually made more convergent or divergent.

### The X-Rays in Ophthalmic Surgery.

In industrial districts where perforating wounds of the eyeball are of almost daily occurrence an $x$-ray outfit is an essential part of the equipment of an ophthalmic hospital. In the majority of cases it is impossible to determine by ordinary methods of examination whether or not the foreign body that made the wound is still within the eye, consequently before the introduction of radiography

* Previous articles appeared on Nov. 11, 25, Dec. 9, 30, 1911; Jan. 13, 27, Feb. 17, March 9, 30, April 20, May 4, 25, June 8, July 6, Aug. 3, 17, 31, Sept. 28, Oct. 12, 26, Nov. 9, 30, Dec. 14, 1912; Jan. 11, Feb. 1, March 1, April 12, and May 3, 17, 31.

† 75 New Cavendish Street, W.

it often happened that small foreign bodies were missed—with disastrous results to the eye; and, on the other hand, eyes with no foreign body in them were often unnecessarily interfered with in the attempt to arrive at a diagnosis.

By means of the $x$-rays it is now a simple matter to discover the presence of, and accurately localise, a metallic foreign body in the eye, even if it measures less than $\frac{1}{2}$ mm. in diameter and weighs less than $\frac{1}{2}$ grain, as several have done in the present writer's experience. The removal of a foreign body by a magnet or other means is, of course, greatly facilitated when its size, shape, and position have been demonstrated by the $x$-rays, but the value of the rays is even greater when they enable us to say definitely that there is no foreign body present, for then the patient can be put to bed and kept quiet without the necessity of further tampering with the damaged eye.

The method of localisation described by Holth, of Christiania ("Ophthalmoscope," Vol. IX., p. 550), has in the present writer's hands proved satisfactory and simple. Two preliminary radiograms are taken and if a foreign body is found to be present the following procedure is adopted. Two small lead discs (2 mm. in diameter) are stitched to the conjunctival limbus, above and below the cornea respectively in the vertical meridian, and their shadows act as landmarks in the radiograms. Two localising radiograms are now taken—one occipito-frontal, the other bi-temporal—and the distances between the shadows of the discs and that of the foreign body are accurately measured in millimetres on both plates. The measurements are then marked by two ink spots on a specially designed metal model of an eye, and from this data it is a simple matter to localise the position of the foreign body in the patient's eye.

The only disadvantage of the method is that some time is taken up in suturing the lead discs to the limbus. To obviate this the present writer is experimenting with very small metal clips which can be attached to the limbus by means of a special pair of pressure forceps, after the manner of Michel's sutures. Should this plan prove satisfactory further details will be published in due course, together with particulars of a special stand for fixing the patient's head while the radiogram is being taken.

### Electro-Magnets.

An electro-magnet is now found in nearly all ophthalmic departments, and, fortunately, the great majority of foreign bodies that penetrate the eyeball consist either of iron or steel, and, consequently, are removable by means of the magnet. In fact, out of considerably more than 100 cases of penetrating foreign body dealt with at the Durham County Eye Infirmary during the last three years, two only were non-magnetic—one was a piece of copper, the other a lead pellet.

No hard-and-fast rule can be laid down for the treatment of magnet cases; each one must be dealt with according to the special features which it presents, but the *first principle* must always be to remove the foreign body as *quickly as possible*. The patient should be $x$-rayed as soon as he presents himself, and while he is being prepared for operation the plates should be developed. If the negatives show a large foreign body in the eye there is no need to waste time in localising it further; but if the foreign body be small, or there is reason to believe that it is non-magnetic, it must be localised by some reliable method such as the one just described.

If two preliminary radiograms are taken, one with the patient looking upwards as far as possible and the other with him looking down to the ground, it is generally possible, by comparing the positions of the shadows of the foreign body with fixed bony points in the orbit, to say definitely whether or not the foreign body is actually in the eyeball itself or whether it has passed through the eye and is lying in the orbit. If there is still any doubt localising buttons must be used and the position of the foreign body accurately calculated.

Two magnets should be at hand—a giant magnet, such as Haab's, capable of supporting a weight of 600 lbs. or more, and a large hand magnet (Hirschberg's is very convenient to use) which will carry about 30 lbs. Both kinds can be wound for use directly on the electric lighting supply, or failing this a fairly powerful magnet can be obtained that will work with a large bichromate battery or a battery of accumulators.

If the foreign body be a large one it should be removed with a small magnet; in fact, it would be disastrous to drag it out rapidly with the large magnet as a large part of the uveal tract would probably come as well. A small foreign body will require the large magnet, and even then a considerable amount of patience must often be exercised before it shows any sign of moving.

When the entrance wound is situated in the sclerotic and the foreign body lies in the vitreous it should be removed through the original wound. If the foreign body has penetrated the cornea and has passed through the lens into the vitreous it can often be drawn into the anterior chamber by means of the large magnet. By judicious manipulation this can be done without further injury to lens or iris, for a foreign body under magnetic influence has a remarkable tendency to slip round the back of the lens and pass through the zonule into the anterior chamber. Once in the anterior chamber it can be very easily coaxed out of the corneal wound with the small magnet.

This section concludes the series of articles,* "Electricity in Modern Medicine." It is, of course, impossible and unnecessary to deal completely with so large a subject in a series of short articles such as these. I have, therefore, attempted to emphasise some elementary practical points that are usually taken for granted in the text-books, for, one finds, it is these very points that beginners so frequently have difficulty in grasping.

[The End.]

* The whole series will shortly be published in book form by The Scientific Press, Ltd.

